# Comparative Metabolomics Reveals Family–Genus-Specific Chemical Signatures and Potential Recognition Mechanisms in *Cynomorium songaricum*–Host Interactions

**DOI:** 10.3390/molecules31030491

**Published:** 2026-01-30

**Authors:** Yu Tang, Changmao Chen, Xunchao Zhang, Yubi Zhou, Jie Wang

**Affiliations:** 1 School of Pharmacy, Qinghai Minzu University, Xining 810007, China; 17355380151@163.com (Y.T.); 18388405563@163.com (C.C.); 2 Qinghai Provincial Key Laboratory of Tibetan Medicine Pharmacology and Safety Evaluation, Northwest Institute of Plateau Biology, Chinese Academy of Sciences, Xining 810008, China; zhangxunchao24@mails.ucas.ac.cn; 3 University of Chinese Academy of Sciences, Beijing 100049, China

**Keywords:** *Cynomorium songaricum*, host plants, root volatiles, GC–MS, parasitic plant recognition

## Abstract

*Cynomorium songaricum* is an important medicinal holoparasitic plant in the arid regions of northwestern China. Its extremely low seed germination rate relies on chemical signals released from the rhizosphere of specific host plants. This study aimed to elucidate the chemical basis of host recognition by *C. songaricum* by characterizing and comparing the rhizosphere volatile metabolomes of five host plant species. Gas chromatography–mass spectrometry (GC–MS) was used to analyze the rhizosphere volatiles of three *Nitraria* species (*N. roborowskii*, *N. sibirica*, *N. tangutorum*), *Peganum multisectum*, and *Zygophyllum xanthoxylum*. Multivariate statistical analyses, including PCA, HCA, and OPLS-DA, were employed to identify shared and differential metabolites. A total of 116 volatile compounds were identified. Alkanes were the predominant metabolite class (accounting for 46.27–76.47% in each host), and 11 C_11_–C_16_ alkanes were shared by all species. Notably, *Z. xanthoxylon* (belonging to a different family) exhibited a distinct metabolic profile, with a significantly higher proportion of benzene derivatives (35.82%) compared to the other hosts. PCA and cluster analysis revealed family/genus-specific clustering patterns, with *Z. xanthoxylon* forming a separate cluster. Several differential metabolites unique to *Z. xanthoxylon* possessed antimicrobial and stress-resistant activities. This study reveals the chemical signatures of *C. songaricum* host roots. The shared alkanes are proposed as potential background signals for general recognition, though this hypothetical role requires experimental validation. Family/genus-specific compounds might be involved in host selection. The unique chemical profile of *Z. xanthoxylon* suggests that *C. songaricum* may employ a flexible recognition strategy, enabling it to parasitize plants across different families. These findings provide fundamental data for understanding the chemical basis of host–parasite interactions in this species.

## 1. Introduction

*Cynomorium songaricum* Rupr. is a rare perennial holoparasitic herb distributed in the desert ecosystems of northwestern China and also found in the arid desert regions of Central Asia. As a keystone species in arid zones, it holds significant ecological value [1,2]. Simultaneously, its dried fleshy stem is a precious tonic in traditional Chinese medicine, commonly used for nourishing the kidneys, strengthening Yang, and relieving constipation. Modern pharmacological studies have shown that *C. songaricum* is rich in polysaccharides, flavonoids, and terpenoids, exhibiting notable anti-fatigue, antioxidant, and immunomodulatory activities [3,4,5].

Despite its dual ecological and medicinal importance, *C. songaricum* has been listed as Vulnerable (VU) on the IUCN Red List of Threatened Species and is classified as a National Grade II Protected Wild Plant in China [6]. Under conditions lacking specific host-derived chemical signal induction, the seed germination rate of *C. songaricum* is extremely low, which has become a core bottleneck restricting its natural population regeneration [7,8]. Field studies confirm that *C. songaricum* primarily parasitizes plants of the genus *Nitraria* (Nitrariaceae), with occasional hosts including *Peganum multisectum* of the genus *Peganum* and *Zygophyllum xanthoxylum* of the family *Zygophyllaceae* [9,10,11].

The interaction between parasitic plants and their hosts is a classic example of species co-evolution. In root parasitic plant interaction systems, specific volatile compounds released by host roots can act as chemical signals, triggering seed germination in the parasitic plant. Extensive research has confirmed that strigolactones can induce seed germination in plants of the genus Striga, while sesquiterpene lactones can activate germination in plants of the genus *Orobanche* [12,13,14]. These findings highlight the crucial mediating role of root volatiles in belowground chemical communication among plants. However, current research on the seed germination induction mechanism of *C. songaricum* has largely focused on animal-mediated seed dispersal pathways and endophytic fungal symbiosis, leaving a significant research gap regarding the systematic screening and identification of volatile signaling compounds from host roots [9,15]. Furthermore, beyond acting directly as germination signals, root volatiles can also regulate the rhizosphere microenvironment through antimicrobial and allelopathic effects, thereby indirectly influencing host recognition and colonization by the parasitic plant [16,17].

Based on this, the five host plants selected for this study belong to different taxonomic families and genera and possess distinct ecological adaptability characteristics: the three *Nitraria* species (*N. roborowskii*, *N. sibirica*, *N. tangutorum*) are all desert shrubs adapted to saline–alkali and arid habitats; *Peganum multisectum* is a common perennial herb in desert steppes; and *Z. xanthoxylon* is a super-xerophytic plant with extreme tolerance to severe drought and saline–alkali environments [18]. The taxonomic diversity of the host plants raises a scientific question worthy of in-depth exploration: How does *C. songaricum* achieve the recognition and parasitism of hosts across different genera and families? This study proposes the following hypothesis: *C. songaricum* employs a dual recognition strategy. First, it perceives conserved compounds shared among different host plants to assess host suitability. Second, it recognizes genus-specific characteristic compounds to achieve precise host selection.

To test this hypothesis, this study employed gas chromatography–mass spectrometry (GC–MS) metabolomics to systematically analyze, identify, and compare the root volatile components of the five host plant species. The specific research objectives are as follows: (1) to comprehensively profile the root volatile metabolomes of the five host plants; (2) to clarify the metabolic differences and clustering characteristics among different hosts through multivariate statistical analysis; and (3) to screen for shared and differential metabolites by combining Venn diagram analysis and orthogonal partial least squares–discriminant analysis (OPLS-DA).

By revealing the chemical basis of the interaction between *C. songaricum* and its hosts, this study aims to provide a critical theoretical and technical foundation for the artificial cultivation and population restoration of *C. songaricum*, while also offering new insights into the chemical ecology of root parasitic plant–host interactions.

## 2. Results

### 2.1. Qualitative and Quantitative Analysis of Volatile Metabolites

To clarify the chemical composition of root volatiles from the five host plants of *C. songaricum*, this study systematically analyzed these compounds using gas chromatography–mass spectrometry (GC–MS). The results showed a total of 116 volatile compounds were identified (see Table S1 in Supplementary Material). The total ion chromatograms indicated that the detection times for these volatile components were concentrated within the range of 4–26 min (see Figure S1 in Supplementary Material). The number of compounds detected varied markedly among the different hosts, ranging from 34 to 67: *Nitraria sibirica* (NS) had the fewest detected compounds (34), while *Z. xanthoxylon* (ZX) had the most (67). The numbers for *N. roborowskii* (NR), *N. tangutorum* (NT), and *P. multisectum* (PM) were 41, 52, and 40, respectively.

**Figure 1:**
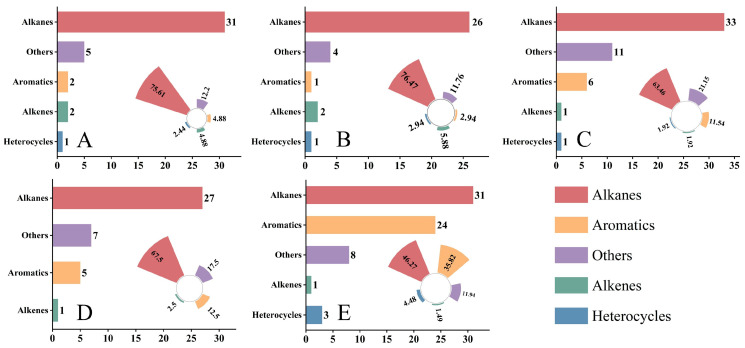
Number and relative proportion distribution of various volatile compound classes in different samples. Data presented in this figure were obtained from volatile component detection of five host plant species listed in the table, and bar charts as well as radial bar charts were generated using Origin 2021 software. Panels (**A**–**E**) correspond to species 1–5 in the table (**A**): *Nitraria roborowskii* Kom. (NR); (**B**): *Nitraria sibirica* Pall. (NS); (**C**): *Nitraria tangutorum* Bobrov (NT); (**D**): *Peganum multisectum* (Maxim.) Bobrov (PM); (**E**): *Zygophyllum xanthoxylum* (Bunge) Maxim. (ZX), respectively. In each panel, the left bar chart indicates the detected number of compounds categorized as Alkanes, Aromatics, Alkenes, Heterocycles, and Others, while the right radial bar chart represents the relative proportion of each compound class in the total detected compounds of the corresponding species. For statistical considerations, three technical replicates were set for each host plant species, and the values of compound number and proportion in the figure are the mean values of three replicates.

The identified compounds were classified into different chemical categories (Figure 1). Alkanes were the most abundant volatile class across all five hosts: in NR, NS, NT, PM, and ZX, the numbers of alkanes were 31, 26, 33, 27, and 31, accounting for 75.61%, 76.47%, 63.46%, 67.50%, and 46.27% of the total identified compounds in each host, respectively. Aromatic hydrocarbons and alkenes were detected in all samples, but at relatively low proportions. Notably, ZX exhibited 24 aromatic hydrocarbons and 3 alkenes, corresponding to proportions of 35.82% and 11.94%, respectively, which were significantly higher than those in the other four hosts.

### 2.2. Global Metabolomic Differentiation Among Host Species

To assess the overall differences in the rhizosphere volatile metabolomes of the five host plants, principal component analysis (PCA) and hierarchical cluster analysis (HCA) were performed based on the relative contents of all identified metabolites.

**Figure 2:**
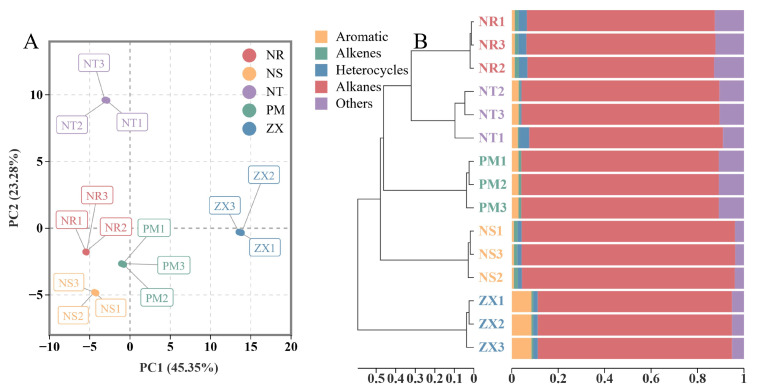
Principal component analysis (PCA) and sample clustering of volatile metabolite profiles in different host plant samples. Panel (**A**) is the PCA score plot, and Panel (**B**) is the sample cluster bar plot; data were obtained from GC-MS analysis of volatile metabolites in five host plant species of *C. songaricum* (three technical replicates per composite sample). The PCA was performed using R version 3.5.1 (stats package, version 3.5.1), with log2 transformation and UV normalization applied to the data before analysis, respectively, and points with different colors/shapes correspond to samples of other species (NR: *Nitraria roborowskii*; NS: *Nitraria sibirica*; NT: *Nitraria tangutorum*; PM: *Peganum multisectum*; ZX: *Zygophyllum xanthoxylum*). The cluster bar plot was generated using R version 4.2.0 (ggplot2 package, version 3.3.6), with Bray distance used for cluster distance calculation; data in the bar plot of Panel (**B**) are the normalized results of compound peak areas, which reflect both the clustering similarity among samples and the compound composition distribution within each sample.

The PCA score plot showed clear separation of the samples from the five hosts in the two-dimensional space (Figure 2A). The first principal component (PC1) explained 45.35% of the total variance, and the second principal component (PC2) explained 23.28%, resulting in a cumulative contribution rate of 68.63%. Replicate samples from the same host clustered closely in the PCA plot, indicating good reproducibility of the measurements. Along the PC1 axis, the four species from the Nitrariaceae family (NR, NS, NT, PM) were grouped, suggesting high similarity in their metabolic profiles. In contrast, ZX from the Zygophyllaceae family was located in a different quadrant, indicating that its metabolome differed from those of the Nitrariaceae hosts. Along the PC2 axis, NT was distributed in another quadrant.

The HCA results further clarified the clustering relationships among the samples (Figure 2B). The samples were primarily divided into three main clusters: one cluster contained the three Nitrariaceae species (NR, NS, PM), which also showed close branching distances in the dendrogram, further confirming the similarity of their metabolic profiles. The other two clusters consisted of NT and ZX separately, with ZX exhibiting the farthest branching distance from all other samples. These results indicated a certain degree of similarity in volatile metabolite composition among the Nitrariaceae hosts, while ZX displayed a unique metabolic profile distinct from all other hosts. Combined with the chemical composition shown in Figure 2B, the significantly higher proportion of aromatic hydrocarbons in ZX was likely one of the key factors contributing to its separation from the other hosts in both PCA and HCA.

### 2.3. Shared Volatile Compounds Among Host Species

**Figure 3:**
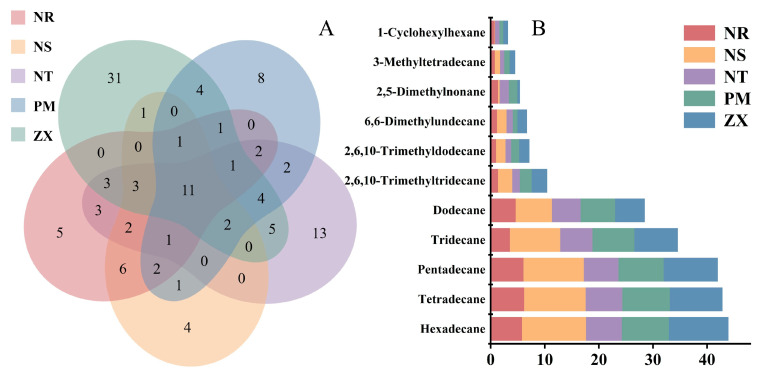
Venn diagram analysis of volatile compounds and stacked bar plot of peak areas for shared components in different host plants. Panel (**A**) is a Venn diagram, and Panel (**B**) is a stacked bar plot of shared components; data were derived from volatile compound detection results of five host plant species (NR: *Nitraria roborowskii*; NS: *Nitraria sibirica*; NT: *Nitraria tangutorum*; PM: *Peganum multisectum*; ZX: *Zygophyllum xanthoxylum*). The Venn diagram was generated using R version 3.5.1 (VennDiagram package, version 1.6.20), which illustrates the shared and unique volatile compounds among different host plants, with values in the diagram representing the number of compounds in the corresponding sets (species). The stacked bar plot was created using Origin 2021 software, showing the relative content distribution of shared volatile compounds across different host plants; different colors in the plot correspond to other host plants, the *y*-axis represents compound names, and the *x*-axis represents the normalized relative content of peak areas.

To identify potential germination-signaling shared compounds among the root volatile components of the five host species, this study performed Venn diagram analysis to examine the shared and unique volatile constituents across the hosts. The results revealed that, out of the 116 identified compounds, 11 were common to all five hosts (Figure 3A). The Venn diagram also indicated the presence of unique volatile components in each host: for example, NR had 5 unique compounds, NS had 4, NT had 13, PM had 8, and ZX had 31, further highlighting the distinctiveness of their metabolic profiles.

All shared compounds were identified as C_11_–C_16_ alkanes, including 2,5-dimethylnonane, hexylcyclohexane, n-dodecane, n-tridecane, n-tetradecane, and others (Figure 3B). As shown in Figure 3B, shared alkanes such as n-dodecane, n-tridecane, and n-pentadecane were present at certain abundance levels in all five hosts. Overall, a trend of increasing abundance with longer carbon chains was observed for some alkanes (e.g., n-hexadecane showed higher abundance in ZX). Notably, n-tetradecane was the most abundant compound across all five hosts, with relative contents of 5.77%, 11.88%, 6.58%, 8.74%, and 10.98% in NR, NS, NT, PM, and ZX, respectively. These widely present and relatively abundant shared alkanes provide fundamental data for exploring the cross-species parasitic mechanism of *C. songaricum*.

Combined with previous chemotaxonomic findings, the unique components likely include categories other than alkanes (for instance, the unique compounds in ZX may be related to its high proportion of aromatic hydrocarbons).

### 2.4. Differential Volatile Metabolites Among Host Species

**Figure 4:**
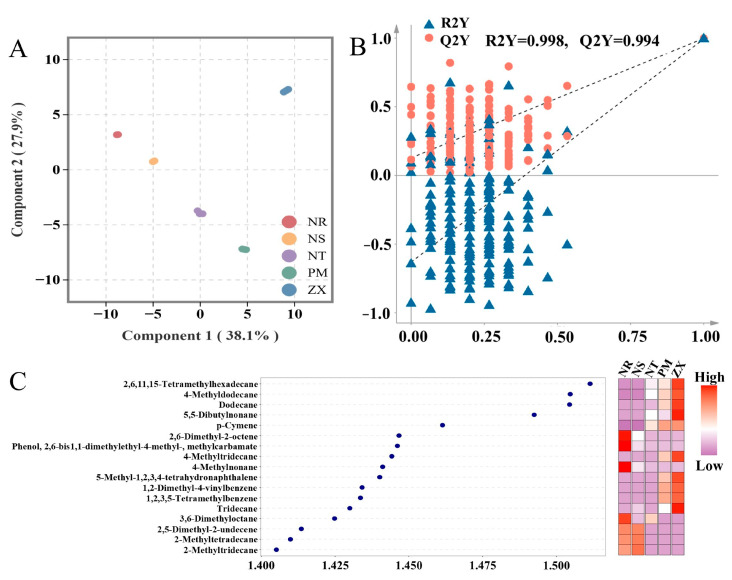
OPLS-DA analysis, permutation test, and VIP value-heatmap of volatile metabolites in different host plants. Panel (**A**) is the OPLS-DA score plot, Panel (**B**) is the permutation test plot, and Panel (**C**) is the VIP value plot combined with a heatmap. Data were obtained from GC-MS analysis of volatile metabolites in five host plant species of *C. songaricum* (three technical replicates per composite sample). (NR: *Nitraria roborowskii*; NS: *Nitraria sibirica*; NT: *Nitraria tangutorum*; PM: *Peganum multisectum*; ZX: *Zygophyllum xanthoxylum*). Both OPLS-DA analysis and permutation test were performed using R version 3.5.1 (MetaboAnalystR package, version 1.0.1). Before analysis, the data were processed with log2 transformation and UV normalization. Zero values were replaced with half of the minimum positive value in the corresponding group. Points of different colors correspond to samples of various species. Panel (**B**) shows the results of 200 permutation tests. The VIP value plot-heatmap was generated using R version 4.2.0 (ggplot2 package, version 3.3.6). In Panel (**C**), VIP values are used to evaluate the contribution of metabolites to inter-group differences, and the heatmap displays the relative content distribution of the corresponding metabolites across different samples (color intensity indicates content level).

To identify volatile metabolites with significant content differences among the hosts, orthogonal partial least squares–discriminant analysis (OPLS-DA) was performed. In the OPLS-DA score plot of the model, the first principal component explained 38.1% of the variance, and the second principal component explained 27.9%, with clear separation between groups observed (Figure 4A). A permutation test with 200 iterations showed no crossover in the fitting curves of R^2^Y (0.998) and Q^2^Y (0.994), and the Q^2^Y intercept was far below zero, confirming that none of the models were overfitted (Figure 4B). This stability and reliability provided theoretical support for the screening of differential metabolites.

Based on the criterion of variable importance in projection (VIP) values greater than 1.4, 17 differential volatile metabolites were screened, including Hexadecane, 2,6,11,15-tetramethyl-; Dodecane, 4-methyl-; Dodecane; 5,5-Dibutylnonane; p-Cymene, and others. Alkanes constituted the majority of these differential metabolites, which is consistent with the earlier chemical classification results (Figure 4C). The heatmap further indicated that high-content differential metabolites were mainly concentrated in ZX (10 metabolites) and NR (7 metabolites). Moreover, the relative abundances of differential metabolites in ZX, such as Hexadecane, 2,6,11,15-tetramethyl-, were significantly higher than in other hosts, while those in NS, NT, and PM were predominantly at low to medium abundance levels.

## 3. Discussion

### 3.1. Family-Specific Metabolomic Profiles Reflect Phylogenetic Relationships

It is important to note that the proposed signaling role of these shared alkanes remains hypothetical and is solely based on correlative metabolomic evidence from the present study; direct biological validation via germination assays is thus required to confirm this function. This study found that the root volatile metabolomes of the five host plants of *C. songaricum* were predominantly composed of alkane compounds, which aligns with the overall chemical profile of plant root exudates [19]. Alkanes are core components of plant cuticular wax, and their enrichment in the root system is presumed to originate from the synthesis and secretion processes of root epidermal cells [20,21]. This characteristic represents a key adaptive trait for plants in arid environments, serving both to reduce water loss and to strengthen physical defense capabilities [22].

In this study, the metabolomic profiles of different samples exhibited significant differentiation. Results from PCA and HCA showed that the five host species could be clearly distinguished based on their volatile metabolomic features, and the clustering pattern was largely consistent with their phylogenetic relationships [23,24]. The four species belonging to the Nitrariaceae family clustered together preferentially, demonstrating higher metabolic similarity, whereas *Z. xanthoxylon*, which belongs to a different family (Zygophyllaceae), formed an independent branch with a unique metabolic fingerprint. This genus-specific metabolic differentiation is of great significance for deciphering the host recognition mechanism of *C. songaricum*—its ability to distinguish the metabolomic profiles of plants from different genera suggests that it may have evolved a sophisticated chemical recognition system. This system would enable it to identify suitable hosts across taxonomic boundaries while maintaining a certain degree of host specificity [25,26].

### 3.2. Shared Alkanes as Potential Universal Recognition Signals

A key finding of this study was the detection of 11 shared alkane compounds (C_11_–C_16_) across all five host plants, with the presence of these compounds unaffected by differences at the genus or family level. These shared compounds include tetradecane (the most abundant single compound in all hosts), dodecane, tridecane, and hexylcyclohexane. They may collectively constitute a “chemical background signal” that conveys the message of “suitable host presence” to *C. songaricum*.

The potential signaling function of these alkane compounds is supported by their known biological properties. Previous studies have indicated that most of these shared alkanes are involved in plant cuticular wax biosynthesis and also possess antimicrobial activity [27,28]. This “dual function” holds significant ecological importance: healthy hosts continuously synthesize cuticular wax and release such alkanes, thereby providing *C. songaricum* with a chemical indicator reflecting “host vitality and suitability.”

This study proposes that the host recognition strategy of *C. songaricum* may differ from that of other parasitic plants like Striga. Unlike the traditional mechanism reliant on single germination stimulants such as strigolactones [29,30], *C. songaricum* may achieve broad-spectrum host recognition by perceiving a composite alkane signal, thereby facilitating seed germination [31,32]. This strategy would confer a significant survival advantage for a generalist parasitic plant capable of infecting hosts across different genera and families.

### 3.3. Genus-Specific Compounds and Host Selection

While the shared alkanes may provide *C. songaricum* with general cues for host recognition, the genus-specific differential compounds identified in this study are likely involved in a more refined host selection process. The most prominent example is *Z. xanthoxylon*: its metabolomic profile differed significantly from the other hosts, with a distinguishing feature being the remarkably high proportion of benzene derivatives at 35.82%, compared to only 4.88–12.50% in the other hosts.

Most genus-specific compounds, particularly those unique to *Z. xanthoxylon*, have reported functions related to “self-defense” and “mediating allelopathic effects.” Among them, benzene derivatives have been confirmed to possess strong antimicrobial activity [33]. They can enhance resistance against pathogens and pests, while also inhibiting seed germination and root growth of competing neighboring plants, thereby improving the host’s own survival competitiveness [34,35]. Alkane compounds unique to Z. xanthoxylon—such as the long-chain and branched alkanes 2,6,11,15-tetramethylhexadecane and 4-methyldodecane—are also implicated in plant self-defense and cuticular wax-layer synthesis [36,37]. This reflects the greater diversity and enrichment of root volatile components in Z. xanthoxylon as a super-xerophytic species [38].

From an ecological perspective, the enrichment of compounds in *Z. xanthoxylon* is particularly noteworthy. As a super-xerophytic plant adapted to extremely arid environments, *Z. xanthoxylon* likely possesses a well-developed chemical defense system [18]. The fact that *C. songaricum* can successfully parasitize this host with a “well-established defense mechanism” suggests that it may have evolved relevant mechanisms to “tolerate or suppress host defense responses,” a process potentially mediated by the recognition of specific chemical signals.

Regarding the fate of host plants after parasitism by *C. songaricum*, our field observations indicate that this holoparasitic interaction does not typically lead to immediate host mortality. Instead, hosts often exhibit recurrent parasitism in subsequent growing seasons. This suggests that *C. songaricum* establishes a persistent but non-lethal relationship with its hosts, likely causing reduced vigor and altered resource allocation rather than rapid death [39]. We speculate that such a potential long-term parasitic strategy could represent an adaptive evolution to resource-scarce desert environments. Accordingly, the chemical recognition mechanisms identified in this study are hypothesized to not only potentially aid in the initial host location by the parasitic plant but also might facilitate the establishment and maintenance of a relatively stable perennial parasitic relationship with the host, thereby possibly contributing to a balanced interaction between the two [40]. It should be emphasized that this hypothetical framework awaits validation through future experimental biological studies.

### 3.4. Limitations and Future Perspectives

A limitation of this study is that all conclusions are based on correlative metabolomic analyses; the proposed signaling functions of the identified alkanes and specific components require direct validation through biological assays. Furthermore, the GC–MS technique primarily focuses on low-boiling-point volatile compounds, potentially offering limited coverage for polar or less volatile potential signaling molecules, such as strigolactones. Future research should focus on: (1) Utilizing artificially collected host root exudates or synthetic standards in strictly controlled bioassays for *C. songaricum* seed germination to verify the individual and combined effects of these shared and unique components; (2) Integrating transcriptomics or other molecular techniques to reveal changes in the expression of genes related to germination pathways (e.g., hormone signal transduction) in *C. songaricum* seeds upon exposure to these potential signaling molecules [41,42,43], thereby confirming their function at the molecular level.

## 4. Materials and Methods

### 4.1. Plant Material

In May 2022, during the seed germination stage of *C. songaricum*, root samples of five different host plants parasitized by *C. songaricum* were collected from Qinghai Province, China. The host plants were identified as parasitized by *C. songaricum* based on visible attachment of *C. songaricum* individuals to their root systems in the field. For each host species, five healthy mature individual plants were sampled. The entire root system was carefully excavated using stainless steel shovels to maintain its integrity and the attached soil. After excavation, loose soil was gently shaken off, and the roots were rinsed with distilled water using soft brushes to remove residual soil and impurities. The cleaned roots were blotted dry with sterile filter paper, then immediately sealed, labeled, and stored in dry ice for prompt transport to the laboratory, where they were ultimately preserved at −80 °C for future use.

All specimens were identified by Researcher Wu Yuhu from the Northwest Plateau Institute of Biology, Chinese Academy of Sciences, and are deposited in the Tibetan Plateau Biological Specimen Museum, Chinese Academy of Sciences. The collection numbers for P1 to P5 range from 391,356 to 391,352, respectively, and their corresponding host plant information is detailed in Table 1.

### 4.2. Instruments and Reagents

The gas chromatography–mass spectrometry system used was the 8890-7000D model (Agilent Technologies Inc., Santa Clara, CA, USA). The electronic analytical balance was of the SOP type (Sartorius Scientific Instruments Co., Ltd., Göttingen, Germany), and the rotary evaporator was model R202-2 (Shanghai Xiafeng Industrial Co., Ltd., Shanghai, China). The reagents included methanol (GC-grade, Merck, Darmstadt, Germany), petroleum ether (60–90 °C) (GC-grade, Tianjin Comio Chemical Reagent Co., Ltd., Tianjin, China), and cyclohexane (GC-grade, Tianjin Comio Chemical Reagent Co., Ltd., Tianjin, China).

### 4.3. Extraction and Concentration of Volatile Compounds

Root samples of each host plant were ground into a fine powder in liquid nitrogen, homogenized thoroughly, and then 100 g of the homogenate was accurately weighed and transferred into a round-bottom flask, dissolved in 300 mL of methanol, and reflux-extracted for 2 h. After the filtrate was collected, the residue was subsequently re-extracted twice with 200 mL of methanol for 1 h and 30 min, respectively. The filtrates from the three extractions were combined and extracted with 100 mL of petroleum ether (60–90 °C) in several portions using a separatory funnel. The petroleum ether layers were collected, evaporated to dryness, dissolved in 5 mL of cyclohexane, filtered through a 0.22 μm microporous membrane, and transferred into a sampling vial for GC-MS analysis. All the above procedures were performed in three technical replicates.

### 4.4. GC Conditions

The Agilent^®^ (Agilent Technologies, Inc., Santa Clara, CA, USA) quartz capillary column model 122-5532 UI, DB-5MS UI (30 m × 0.25 mm × 0.25 μm), was used. The temperature program was as follows: initial temperature 40 °C held for 1 min; heated at 40 °C/min to 120 °C, held for 2 min; heated at 5 °C/min to 240 °C, held for 8 min; and heated at 12 °C/min to 300 °C, held for 10 min. The carrier gas was high-purity nitrogen (purity ≥ 99.999%) or high-purity helium (purity ≥ 99.999%) at a flow rate of 1.0 mL/min. The inlet temperature was 280 °C, and the injection volume was 1.0 μL.

### 4.5. MS Conditions

The MS module was equipped with an electron impact source (EI) that was set at 70 eV. The ion source temperature was 230 °C, and the GC-MS interface temperature was 280 °C. Compounds were identified by comparing mass spectra with the NIST14.L mass spectral library (National Institute of Standards and Technology, Gaithersburg, MD, USA).

### 4.6. Statistical Analysis

All statistical analyses were performed using SPSS Statistics 26.0 software, and graphs were generated using Origin 2021 and R packages (using R version 3.5.1 and R version 4.2.0). Before data analysis, the raw peak area data were subjected to log2 transformation and UV normalization, with zero values replaced by half of the minimum positive value within the corresponding group. For each host species, root samples from five individual plants were pooled in equal quantities to form one composite sample. All subsequent extractions, GC-MS analyses, and multivariate statistical analyses—including PCA, HCA, and OPLS-DA—were based on three technical replicates derived from this composite sample. Specifically, PCA was conducted using the stats package in R version 3.5.1. HCA was performed using the ggplot2 package (version 3.3.6) in R version 4.2.0, with Bray-Curtis distance used to calculate clustering distances. Venn diagram analysis was carried out using the VennDiagram package (version 1.6.20) in R version 3.5.1. OPLS-DA and permutation tests were implemented using the MetaboAnalystR package (version 1.0.1) in R version 3.5.1, and the variable importance in projection (VIP) score heatmap was generated using the ggplot2 package (version 3.3.6) in R version 4.2.0.

## 5. Conclusions

This study employed GC-MS metabolomics to systematically analyze the root volatiles of five host plants of *C. songaricum*. A set of shared C_11_–C_16_ alkanes was identified across all hosts. These alkanes are proposed as candidate universal chemical background signals, enabling *C. songaricum* to identify suitable hosts across taxonomic units, reflecting a flexible strategy distinct from traditional single-molecule recognition.

Furthermore, multivariate analysis revealed family- and genus-specific metabolic differentiation. The four Nitrariaceae species exhibited similar metabolic profiles, while *Z. xanthoxylon* formed a separate cluster characterized by a high proportion of benzene derivatives and unique defensive/stress-resistant compounds. This indicates that *C. songaricum* can distinguish hosts at the family level.

Finally, the biological functions of many specific compounds include antimicrobial activity and involvement in cuticular wax synthesis. This suggests that host defensive substances may have a dual role: defending against pathogens and providing recognition cues for the parasitic plant.

In conclusion, this study identified a set of shared alkanes and proposed a hypothetical dual-recognition strategy for *C. songaricum* that combines putative universal alkane signals with genus-specific markers. This model, derived from chemical profiling, provides a testable framework for future research to validate the biological activity of these candidate signals. These findings provide a foundation for developing artificial germination inducers and support a deeper understanding of co-evolutionary mechanisms in parasitic plant systems.

## Figures and Tables

**Table 1:** Collection of details of host plant samples.

No.	Species	Abbreviation	Producing Area	Geographic Coordinates	Harvest Time
1	*Nitraria roborowskii* Kom.	NR	Delingha City	36.84° N, 98.60° E	8 May 2022
2	*Nitraria sibirica* Pall.	NS	Delingha City	37.31° N, 96.73° E	8 May 2022
3	*Nitraria tangutorum* Bobrov	NT	Delingha City	37.34° N, 97.08° E	8 May 2022
4	*Peganum multisectum* (Maxim.) Bobrov	PM	Guide County	36.06° N, 101.43° E	10 May 2022
5	*Zygophyllum xanthoxylum* (Bunge) Maxim.	ZX	Guide County	36.05° N, 101.44° E	10 May 2022

## Data Availability

The data associated with this study are included in this article and its Supplementary Information Files.

## Supplementary Materials

The following supporting information can be downloaded at: https://www.mdpi.com/article/10.3390/molecules31030491/s1, Table S1 lists the volatile components of the sample roots, and Figure S1 shows the total ion-current chromatogram of the samples.

## Abbreviations

The following abbreviations are used in this manuscript:

EI	Electron Impact Source
GC-MS	Gas Chromatography–Mass Spectrometry
HCA	Hierarchical Cluster Analysis
IUCN	International Union for Conservation of Nature
KEGG	Kyoto Encyclopedia of Genes and Genomes
MS	Mass Spectrometry
OPLS-DA	Orthogonal Partial Least Squares–Discriminant Analysis
PCA	Principal Component Analysis
VIP	Variable Importance in Projection
VU	Vulnerable
