# Supplementary material for: Comparative Metabolomics Reveals Family–Genus-Specific Chemical Signatures and Potential Recognition Mechanisms in Cynomorium songaricum–Host Interactions
Source: Molecules. 2026 Jan 30;31(3):491. doi: 10.3390/molecules31030491 (PMC12898630; doi:10.3390/molecules31030491)
Supplement: Supplementary file 1 [file molecules-31-00491-s001.zip › Figure S1 shows the total ion-current chromatogram of the samples.pdf]

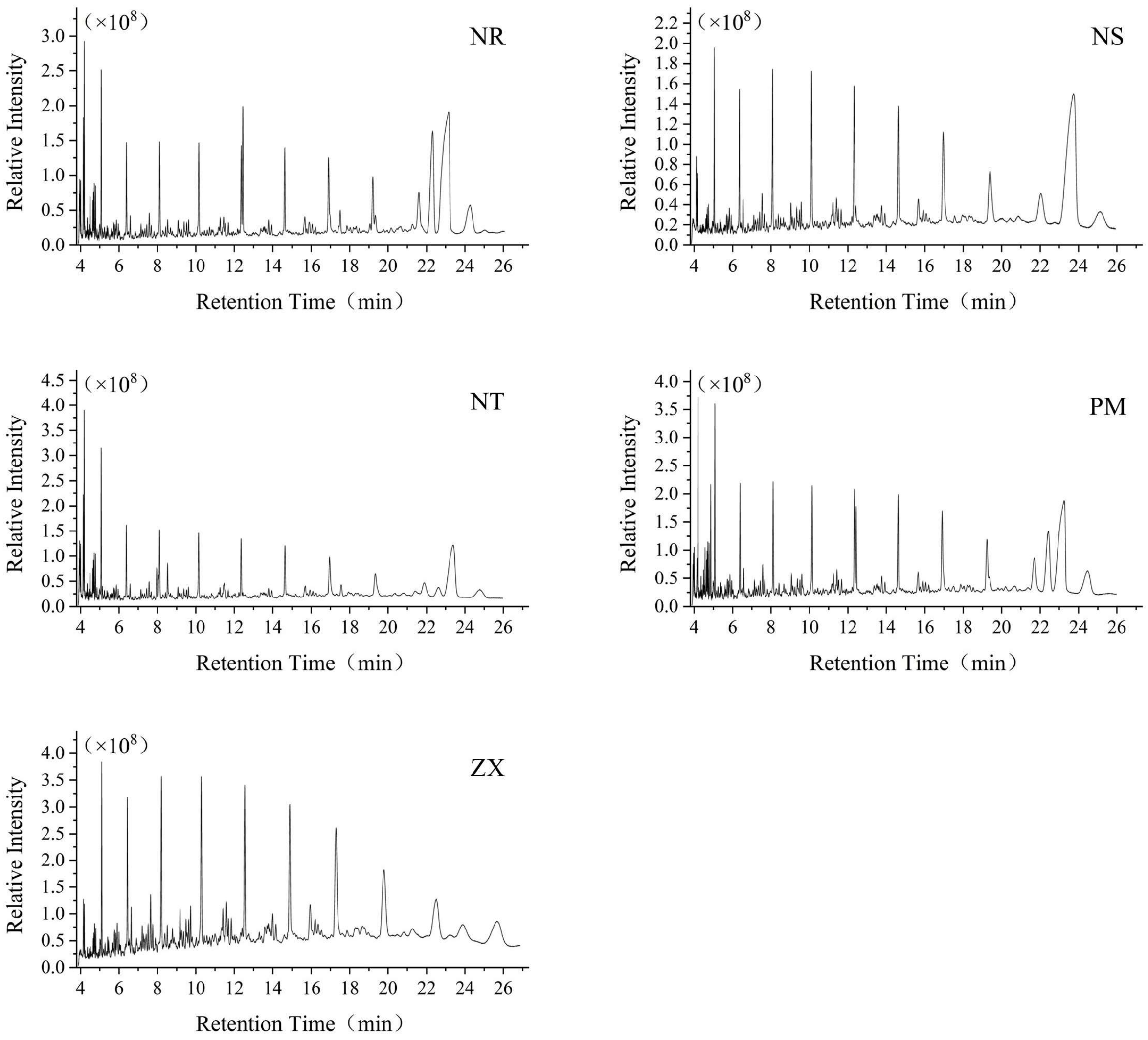

Figure S1 Sample total ion flow diagram

Note: NR: *Nitraria roborowskii* Kom., NS:*Nitraria sibirica* Pall., NT: *Nitraria tangutorum* Bobrov, PM: *Peganum multisectum* (Maxim.) Bobrov, ZX: *Zygophyllum xanthoxylum* (Bunge) Maxim.
